# Signaling Proteins and Transcription Factors in Normal and Malignant Early B Cell Development

**DOI:** 10.1155/2011/502751

**Published:** 2011-05-20

**Authors:** Patricia Pérez-Vera, Adriana Reyes-León, Ezequiel M. Fuentes-Pananá

**Affiliations:** ^1^Laboratorio de Cultivo de Tejidos, Departamento de Investigación en Genética Humana, Instituto Nacional de Pediatría Insurgentes Sur 3700-C. Col. Insurgentes Cuicuilco, 04530 México, DF, Mexico; ^2^Unidad de Investigación Médica en Enfermedades Infecciosas y Parasitarias (UIMEIP), Hospital de Pediatría Centro Médico Nacional Siglo XXI, Instituto Mexicano del Seguro Social, Avenida Cuauhtémoc 330, Colonia Doctores, Delegación Cuauhtémoc, 06720 México, DF, Mexico

## Abstract

B cell development starts in bone marrow with the commitment of hematopoietic progenitors to the B cell lineage. In murine models, the IL-7 and preBCR receptors, and the signaling pathways and transcription factors that they regulate, control commitment and maintenance along the B cell pathway. E2A, EBF1, PAX5, and Ikaros are among the most important transcription factors controlling early development and thereby conditioning mice homeostatic B cell lymphopoiesis. Importantly, their gain or loss of function often results in malignant development in humans, supporting conserved roles for these transcription factors. B cell acute lymphoblastic leukemia is the most common cause of pediatric cancer, and it is characterized by unpaired early B cell development resulting from genetic lesions in these critical signaling pathways and transcription factors. Fine mapping of these genetic abnormalities is allowing more specific treatments, more accurately predicting risk profiles for this disease, and improving survival rates.

## 1. Introduction 

The main function of mature immunocompetent B cells is to make antibodies; therefore, they are responsible for the adaptive humoral immune response. Formation of these mature B cells is a highly ordered multistep process that in adult mammals starts in bone marrow with the commitment of hematopoietic stem cells to the B cell lineage and ends with formation of mature B cells in secondary lymphoid organs. This developmental process was once thought to be inflexible, unidirectional, and irreversible, but recent lines of evidence support a higher level of plasticity to the differentiating cell. Several transcription factors (TFs) play critical roles in commitment and maintenance along the B cell pathway, and their gain or loss affects homeostatic B cell lymphopoiesis and often results in malignant transformation.

## 2. The Early Stages of B Cell Development

**Figure 1:**
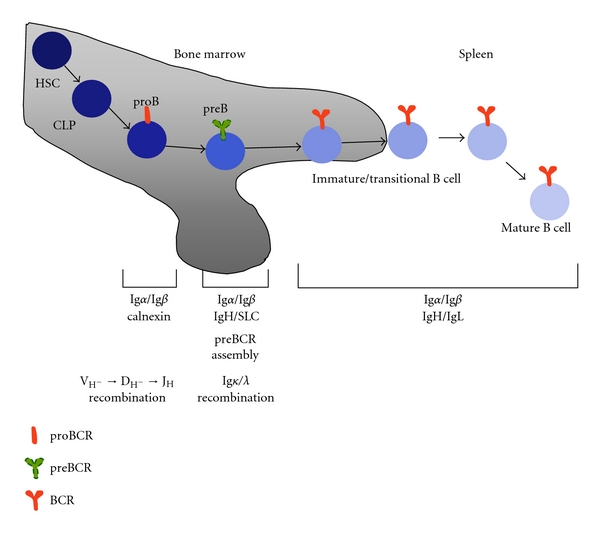
B cell development in adult mammals starts in bone marrow with the commitment of hematopoietic stem cells (HSCs) to the B cell lineage and ends with formation of mature B cells in peripheral secondary lymphoid organs (e.g., the spleen). It is the sequential expression and assembly of the components of the B cell antigen receptor (BCR) what defines each developmental stage. The first stage exhibiting commitment to the B cell lineage is the proB, and here the immunoglobulin heavy chain is in the process of recombination, and the signaling proteins Ig*α* and Ig*β* are in surface forming complexes with chaperon proteins like calnexin (the proBCR). The next developmental stage, the preB, happens after the heavy chain was successfully recombined and the preBCR is assembled. In this stage, the light chain is recombined and unrearranged heavy chain alleles are excluded. After light chain recombination and pairing with the heavy chain and Ig*α* and Ig*β* the mature BCR is formed, the B cell is in the immature (in bone marrow) and transitional (in periphery) stages. Here, B cell mechanisms of self-tolerance are active allowing self- and nonself-recognition by the mature B cell. Transition to the mature stage happens if the BCR of the immature/transitional B cell does not find its cognate antigen after several days of bone marrow and peripheral trafficking.

B cells express receptors (BCRs) to various antigens; each BCR is uniquely specialized to recognize and counteract a particular new or recurrent pathogen. The main goal of B cell development is to generate B cells expressing a diverse repertoire of BCRs. This receptor is a membrane-bound immunoglobulin (Ig) consisting of a heterodimer of identical pairs of the Ig heavy and light chains, which are responsible for the clonal diversity of the B cell repertoire. The Ig heavy and light chain heterodimer is, however, unable to generate signals to trigger biological responses after antigen binding; this function is mediated by the disulfide-coupled heterodimer of Ig*α* (CD79a) and Ig*β* (CD79b), which is noncovalently associated with the Ig antigen recognition unit [1]. It is the sequential expression and assembly of these BCR components that defines each developmental stage, and, therefore, each stage is characterized by a particular form of the BCR, reflecting the progression of receptor assembly (Figure 1) [2, 3].

**Figure 2:**
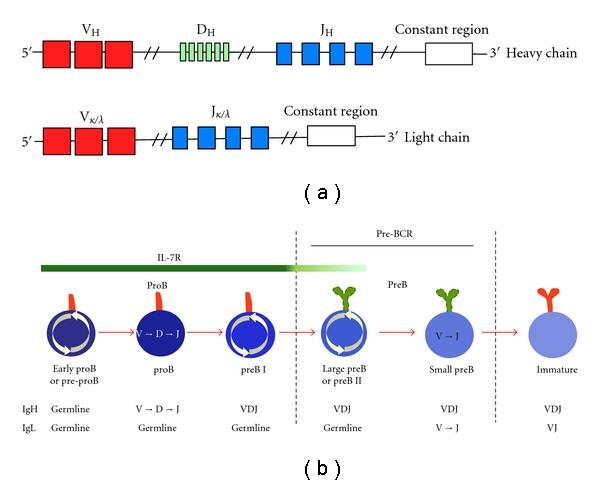
(a) The Ig heavy and light chain genes are comprised of constant and variable regions, where the variable region is formed by an *n* number of segments termed V (variable), D (diversity), and J (joining) in the heavy chain and by segments V and J in the light chain. These segments are brought together by a site-specific recombination process termed VDJ recombination responsible for the extensive repertoire of BCR specificities. There are two loci for light chain, *κ* and *λ*. Here, all the loci are shown in germline configuration, previous to the process of VDJ recombination. (b) The early stages of B cell development are differentiated by the process of VDJ recombination, and the heavy (IgH) and light (IgL) chains are recombined in the proB and preB stages, respectively. Each stage is further subdivided according to the sequential assembly of the VDJ segments. Replication and recombination processes are mutually exclusive as denoted by the circular arrows and VDJ signs inside the cell. Dashed lines separating proB and preB stages indicate checkpoints where signaling from the preBCR and BCR is required for positive selection and progression along the B cell maturation pathway. Continuous lines indicate the main receptors controlling each developmental stage. The differential intensity in the IL-7 green line indicates the sub-stages where a higher or lower concentration of the IL-7R ligand is required.

To achieve BCR clonal diversity, the Ig heavy and light chain genes are composed of constant and variable regions. The variable region is formed by a series of segments termed V (variable), D (diversity), and J (joining) (Figure 2(a)), which are brought together by a site-specific recombination process termed VDJ recombination. This is a highly ordered process during which the D and J fragments are rearranged and the V segment is then joined to the DJ fragment; these steps occur first in the heavy and then in the light chain loci (Figure 2(b)) [4, 5]. This process is essential for the lymphocyte's adaptive immune function. The first developmental stage exhibiting commitment to the B cell lineage is called proB and is characterized by rearrangement of the Ig heavy chain [2]. The proB stage is further divided according to the status of the heavy chain; in mice, Marshall named these substages proB-A (during which the heavy chain is in the germ line state), B (during which D and J are recombined), and C (during which V-DJ is recombined) [6]. These stages are better known in humans as early proB or preproB (A), proB (B) and preB I (C) (Figure 2(b)).

In the proB stage Ig*α* and Ig*β* are expressed at the cell surface in association with chaperon proteins such as calnexin [7]. As soon as the heavy chain is successfully recombined, it is assembled with Ig*α* and Ig*β* and the surrogate light chains *λ*5 and VpreB to form the preBCR. Surface expression of this receptor marks the transition to the preB stage (Figure 1) [5]. Unsuccessful recombination (with heavy chain VDJ fragments that are not in a proper reading frame) or unsuccessful pairing of the preBCR components results in proB cells that are unable to proceed to the preB stage and leads to apoptosis. This observation supports an active signaling role for the preBCR in generating the permissive signal that allows differentiation through the preB stage. In the preB stage, the light chain V and J fragments are recombined, and, again, successful assembly of the mature form of the BCR marks the transition to the immature stage (Figures 1 and 2(b)) [2, 4, 8, 9].

In addition to their VDJ recombination status, all proB and preB stages can be recognized by their patterns of surface markers expression and their proliferative state [6]. Because DNA replication during proliferation is associated with homologous recombination and VDJ rearrangement is associated with nonhomologous recombination, these two processes are mutually exclusive. ProB and preB stages are thus characterized by waves of VDJ recombination followed by waves of proliferation [6, 10]. To achieve this pattern, the expression of the two main enzymes responsible for VDJ rearrangement, the recombinase associated genes 1 and 2 (RAG1 and RAG2), are tightly regulated during the cell cycle; they are highly active in G0 and degraded before entrance into S phase [11–13]. The developing B cell thereby ensures that no events of non-homologous recombination will occur during DNA replication and avoids an increase in the mutation rate. This fine regulatory control separating proliferation and differentiation could explain why the proliferative proB and preB substages are often found to be compromised in B cell acute lymphoblastic leukemia (ALL) and why leukemic cells are often unable to differentiate. In proB, proliferative stages occur in early proB or preproB (before heavy chain recombination) and preB-I (after heavy chain recombination), whereas in preB proliferation occurs during the large preB-II stage (before light chain rearrangement) (Figure 2(b)) [6, 10].

## 3. Regulation of Lineage Commitment: The Critical Role of IL-7R, PreBCR, and Downstream B cell Transcription Factors

Limitation of lineage choice during development is regulated by a combination of signaling pathways and transcription factors (TFs). In mice, the main receptor controlling the proB stage is the IL-7R, which is composed of an *α* chain (IL-7R*α*) and the common cytokine receptor *γ* chain (*γ*c) [14, 15]. Deletion of IL-7R*α* or *γ*c leads to developmental arrest at the early proB stage [16–19]. Loss of IL-7 also affects T cell development; however, this can be overcome by enforced expression of Bcl2, supporting a key role in proT and preT survival [20, 21]. On the other hand, enforced expression of antiapoptotic genes cannot compensate for IL-7R loss in B cell development, indicating additional roles for IL-7 in this lineage [22]. In humans, B cells can still be generated in severe combined immunodeficiency (SCID) patients with mutations in the *IL-7R* gene, suggesting that IL-7 signaling is not as essential for human B cell development [23]. However, a recent study has demonstrated that *in vitro* human B cell production is IL-7 dependent [24].

IL-7 activates three major signaling pathways: (1) JAK-STAT, (2) phosphatidylinositol 3-kinase (PI3K)-Akt, and (3) Ras-Raf-Erk [25]. STAT5 (signal transducer and activator of transcription 5) is the predominant STAT protein activated by IL-7 [25, 26]. STAT5 is a transcription factor that consists of two highly related isoforms, STAT5A and STAT5B; these proteins play redundant roles because the loss of only STAT5A or STAT5B has minor consequences for lymphocyte function [26]. However, when both STAT5s are lost, B cell development is arrested at the early proB stage, indicating that STAT5 is an essential mediator of IL-7 signaling in early B cell development [27, 28]. IL-7/STAT5 signaling also promotes cell survival via the activation of the anti-apoptotic gene *Mc1* [29]. Once the preBCR is expressed, it can take over many of the functions performed by the IL-7 receptor. The preBCR can signal constitutively or when activated by binding to nonpolymorphic ligands; like IL-7R, it activates the PI3K-Akt and Ras-Raf-Erk pathways [8, 30]. It has been shown that proliferation of large preB-II cells is dependent on the preBCR acting in concert with low (picogram) levels of IL-7 (Figure 2(b)) [31]. The *CCND3* gene, which encodes for cyclin D3, is essential for pre-B cell expansion and integrates IL-7R and preBCR signals [32].

Downstream of the IL-7 and preBCR receptors, a handful of TFs have been observed to be critical for commitment to the B cell lineage and early development; these include E2A/TCF3 (immunoglobulin enhancer binding factors E12/E47/transcription factor 3), EBF1 (Early B cell Factor 1), and PAX5 (Paired box 5) [33–35]. Loss of E2A and EBF1 blocks entry into the B cell lineage, and loss of PAX5 redirects B cells into other lineages [35–37]. Thus, there is a hierarchical relationship in the expression of these transcription factors, with some genes regulating entry into the B cell lineage and others regulating commitment to this lineage [36, 38–40]. Ig recombination generally correlates with germline transcription; transcription probably allows changes in the chromatin structure and/or RAGs accessibility [5]. One of the main molecular functions of PAX5 (acting together with E2A, EBF1 and STAT5) is to allow VDJ recombination [41–43]. Ectopic expression of PAX5 and E2A allows VDJ recombination in non-B cells [44, 45]. Also, E2A, PAX5, IKZF1, and RUNX1, among other TFs, are responsible for RAG expression [46, 47]. Therefore, IL-7R signaling fulfills an essential role in early B cell development, with STAT5 participating in the activation of the B cell regulatory genes *E2A*, *EBF1,* and *PAX5* [37].

*E2A* encodes two TFs via alternative splicing, E12 and E47. In mice lacking the *E2A* gene, the B cell lineage is lost, there is no heavy chain recombination, and the expression of the B-cell-restricted genes *EBF1, PAX5, CD79A/B, IGLL1*, and *VPREB1* (*CD179A*) is also affected [48, 49]. The absence of the E47 isoform results in a diminished B cell population, suggesting a significant role for E47 homodimers in differentiation. In contrast, the lack of E12 results in normal but inefficient B cell differentiation [50]. Loss of *EBF1* results in B cell arrest before heavy chain recombination and failure to express the preBCR components Ig*α* and Ig*β* and the surrogate light chains *λ*5 and VPREB [51]. Enforced expression of *EBF1* and *PAX5* is sufficient to overcome the developmental block in mice deficient in E2A, IL-7, or IL-7R*α*, further illustrating the transcriptional hierarchy of the B-cell specific program triggered by IL-7 receptor signaling [37, 52–56].

The *EBF1* promoter is responsive to PAX5, ETS1, and SP11 (PU.1) and together with PAX5 drives the expression of many genes critical for early B cell development and B cell function, including *FOXO1, MYCN, LEF1, BLNK, CD79A* (*MB-1*), *RAG2, CD19,* and *CR2* (*CD21*) [37, 51, 57]. EBF1 also works at an epigenetic level, controlling chromatin structure by recruitment of molecules as RUNX1 and E-proteins [40, 58]. EBF1 expression has been implicated in demethylation of the *CD79A* promoter, and together with PAX5 and other transcriptional activators, it generates genomic regions of active chromatin [59]. PAX5 can be a positive or negative regulator depending on the transcriptional context [60]; it is generally a positive regulator of B-cell-specific genes such as *EBF1*, *CD19*, *MB-1*, *BLNK*, *IGLL5,* and *VPREB1* [34, 61] and a repressor of non B-lineage genes such as *M-CSR*, *NOTCH1,* and *FLT3* [62–64]. Genome-wide transcriptional analysis has identified a plethora of genes targeted by both EBF1 and PAX5, which cooperate to activate the B cell gene expression program and to allow lineage maintenance by inhibiting the expression of genes associated with other developmental programs and hematopoietic progenitors [65, 66]. Because of EBF1 and PAX5-mediated transcription of B-cell-specific genes and repression of genes associated with other lineages, B cell development is unidirectional and perhaps irreversible in homeostatic conditions [34, 36, 59, 63, 67, 68].

Also important for lymphoid development are members of the Ikaros family of TFs, mainly *IKZF1 *(which encodes Ikaros) and *IKZF3* (which encodes Aiolos); both TFs bind to the same consensus sequence as either homodimers or heterodimers playing distinct roles in B and T cell differentiation [69, 70]. Ikaros activates *CD19* and represses genes that are unrelated to the B lineage, such as *CD4* [71]. Expression of *IKZF1* and *IKZF3* is regulated by alternative splicing, which produces long isoforms (Ik-1, Ik-2, Ik-3, Aio-1, Aio-3, Aio-4, and Aio-6) that have at least three zinc fingers and efficiently bind to DNA, and short isoforms (Ik-4, Ik-5/7, Ik-6, Ik-8, Aio-2, Aio-5) that have less than two zinc finger domains, are unable to bind DNA with high affinity, and do not activate transcription; these short forms, therefore, act as dominant negatives [69, 72, 73]. Ikaros is activated in early stages of lymphopoiesis and is required for both early and late events in lymphocyte differentiation. Ikaros and Helios (another member of the Ikaros family; *IKZF2*) have opposite regulatory roles of the expression of *INPP5D* gene encoding the SHIP phosphatase, a BCR signaling negative regulator [74]. Aiolos is not required during the early specification of the B and T lineages but is essential during B cell maturation [75]. Aiolos together with Ikaros act in concert to repress c-Myc expression in large preB cells with concomitant repression and induction of genes *CCND3* and *CDKNIB*, respectively [76], thus promoting preB cell cycle exit and transition to small preBs where light chain recombination occurs. Therefore, Aiolos has a role during B cell commitment and together with Ikaros during B cell maintenance, hence, mice lacking Ikaros lack all lymphoid lineages [77, 78], whereas Aiolos-deficient mice have an increased pre-B cell population and develop B cell lymphomas [75].

## 4. Genetic Abnormalities in Human Pediatric B Cell Acute Lymphoblastic Leukemia

Because all these TFs are critical for early murine B cell development, it is not surprising that abnormalities in these genes have often been found in human B cell acute lymphoblastic leukemia (B-ALL). ALL is the most common childhood cancer and is characterized by impaired early lymphoid development. ALL can be classified as B or T cell ALL; B-ALL is the one most frequently found (83% of ALLs and 30% to 40% of all childhood cancers) [79, 80]. A high percentage of B cell ALL patients have genetic lesions (mostly chromosomal translocations) that are specifically associated with the leukemic cells. In recent years, efforts have focused on the identification of the specific developmental stage where the ALL cells are arrested and the genetic lesions responsible for the leukemia phenotype. These data have helped to classify the disease, stratify the patients into risk groups and design specific therapies that have significantly improved the survival rate [65, 66].

*RUNX1 *(Runt-related transcription factor 1) is a frequent target for chromosomal rearrangements and mutations in ALL. *RUNX1* (*AML1* or *CBF*α*2*) encodes a TF that possesses a Runt domain, which is essential for interaction with CBF*β*, a heterodimer that regulates transcription of genes that are important in hematopoiesis. CBF cooperates to regulate the survival of early pro-B cells and establish a B-lineage-specific transcriptional program [81]. A substantial proportion (25% of children and 2% of adults) of ALL patients present the *ETV6/RUNX1 *fusion as a result of the translocation t(12;21)(p12;q21). In contrast to the normal function of this TF, the chimeric protein recruits corepressors and histone deacetylases to create stable repression complexes at the promoters of RUNX1 target genes [82]. Homozygous mutations in *RUNX1* in mice are lethal, with embryos lacking fetal liver hematopoiesis [83, 84]. This result supports the idea of different roles for this TF in mice and humans and suggests that RUNX1 plays a more profound role at an earlier stage of murine hematopoiesis.

The TF E2A has been found in various chromosomal translocations; among the most common are t(1;19)(q23;p13), which results in the *E2A-PBX1* fusion, and t(17;19)(q22;p13), resulting in *E2A-HLF* (hepatic leukemia factor). These abnormalities are detected in 5-6% and 1% of children ALL, respectively, and are found in less than 5% of adult cases [37, 85]. These fusion products possess properties that the original proteins do not have. E2A-PBX1 consists of the E2A N-terminal TF domain fused to the Hox cooperative motif and homeodomain of C-terminal PBX1. PBX1 modulates the DNA binding activity of specific subsets of Hox proteins, which participate in normal hematopoiesis, and the chimeric protein E2A-PBX1 may disturb Hox activity [37, 85]. E2A-PBX1 is considered a potent transcriptional activator of the *WNT16* gene; because the *WNT* family is widely known to be involved in oncogenesis, impairment of WNT signaling could be a mechanism of leukemia initiation [86]. E2A-HLF encodes a chimeric protein that contains the E2A N-terminal fused to the C-terminal basic leucine zipper of HLF. E2A-PBX1 and E2A-HLF fusion proteins do not bind the same DNA motifs and might regulate different sets of genes, disturbing different processes. E2A-HLF promotes the enhanced survival of early B cells activating expression of the anti-apoptotic TFs SNAI2 (SLUG) and LMO2, accordingly, gene silencing of *LMO2* in an E2A-HLF^pos^ cell line-induced apoptotic cell death [87].

The *MLL *(mixed lineage leukemia) gene is often rearranged in patients with ALL, both *de novo* and therapy-related. Leukemias with MLL translocations can be myeloid or lymphoid and often tend to express both lineage markers, probably indicating a very early multipotent progenitor origin [88, 89]. Today, more than 50 fusions involving *MLL* have been documented. The translocation t(4;11)(q21;q23) that fuses the *MLL* and *AF4* genes is present in the majority of the *de novo* cases and represents up to 80% of the infant, 2% of children older than 1-year old, and 5 to 10% of adult ALLs. MLL possesses a Trithorax domain that participates in the methylation of lysine 4 of histone H3 (H3K4), and this function is associated with transcriptional activation of target promoters; important for leukemogenesis are *HOX* genes and *Meis1*. Hox proteins are a highly conserved homeobox containing family of TFs that regulates cell fate, including hematopoietic lineage decisions [88–90]. Recent data show that MLL and MLL-fusion proteins are recruited to the target genes by the transcriptional elongation factor Paf1c, and this interaction is required to increase the expression of *HOX* genes and transformation of bone marrow cells [90].

Another important translocation is t(9;22)(q34;q11) (also known as Philadelphia chromosome), which fuses the genes *BCR *and *ABL1* and is found in 5% of childhood and 25% of adult B cell ALL. Unregulated expression of STAT5 has been observed to be associated with *BCR-ABL1* [91, 92]. *STAT5* inactivation results in cell cycle arrest and apoptosis of BCR-ABL1^pos^ malignant B cells, and BCR–ABL1 expression is unable to induce leukemia in STAT5^−/−^ mice, supporting an important role for STAT5 in the initiation of BCR-ABL1^pos^ transformation. Genome-wide analysis of B cell ALL has identified mutations in the Janus kinases (*JAK1* and *JAK2*) in up to 10% of patients [92]. Interestingly, patients with *JAK1/2* mutations and patients with the *BCR-ABL1* fusion both have a similar gene expression profile and a high-risk prognosis [91]. JAK1 and 2 are activated after IL-7R engagement and mediate STAT5 activation; most mutations in these proteins occur in the kinase domain, constitutively activating the JAK-STAT signaling pathway. *JAK* gain-of-function mutations are insufficient to confer a transformed phenotype, and it has been demonstrated that 100% of B-ALL cases with *JAK2* mutations overexpress CRLF2 (type I cytokine receptor subunit, also known as thymic stromal lymphopoietin receptor) [66]. CRFL2 forms a heterodimeric complex with the IL-7R and is overexpressed in 15% of B-ALL cases as a result of translocations or intrachromosomal deletions [66, 92]. In a subset of cases, *CRLF2* acquires a Phe232Cys gain-of-function mutation that promotes constitutive dimerization and cytokine-independent proliferation [92]. In agreement with data suggesting an association between CRLF2 and the JAK-STAT signaling pathway in B-ALL, the gene profile associated with CRLF2 over-expression is also highly similar to that found in BCR-ABL1^pos^ B-ALL. In a subgroup of aggressive treatment-resistant B ALL, deregulation of the JAK-STAT pathway is associated with abnormalities in genes such as *IKZF1* and *CDKN2A*/*B* [66, 91, 92].

Genome-wide analysis has also identified abnormalities in *PAX5* and *EBF1* in B ALL [93]. In the case of *PAX5*, these abnormalities are generally (a) point mutations located in exons 2, 3, 4, 5, 6, 7, 8, 9, and 10, most of which are clustered within functional domains or (b) alterations in the expression of splice variants of PAX5 [94, 95]. These alterations have been found in up to 32% of children and 30% of adults with B ALL and in 35% of relapsed cases [93, 96, 97]. Almost 35% of patients with *PAX5* lesions lack expression of the full-length protein and express only short variants [95]. Mullighan et al. reported *PAX5* deletions in 51% of BCR-ABL1^pos^ B ALL cases [91]. However, the specific association between aberrant splicing and *PAX5* mutations with specific genetic subtypes of B ALL is controversial [95]. In addition, *PAX5* (9p11) participates in gene fusions. Currently, five *PAX5* fusions have been identified, with the gene partners *LOC392027 *(7p12.1), *SLCO1B3 *(12p12), *ASXL1 *(20q11.1), *KIF3B *(20q11.21), and *C20orf112 *(20q11.1). All of the resulting chimeric proteins retain the paired domain of the TF, and, as for the previously described deletions, the gene fusions result in lowered expression of *PAX5* and its target genes [98]. *EBF1* alterations are also common in patients with poor outcomes. In a cohort of 45 adolescent and adult ALL cases, deletions in *EBF1* were observed in 4.4% of cases; in relapsed children, *EBF1* deletions are particularly frequent (25%), suggesting a possible contribution of this TF for the development of relapse and a potential prognostic value [65, 66, 97, 99].

**Figure 3:**
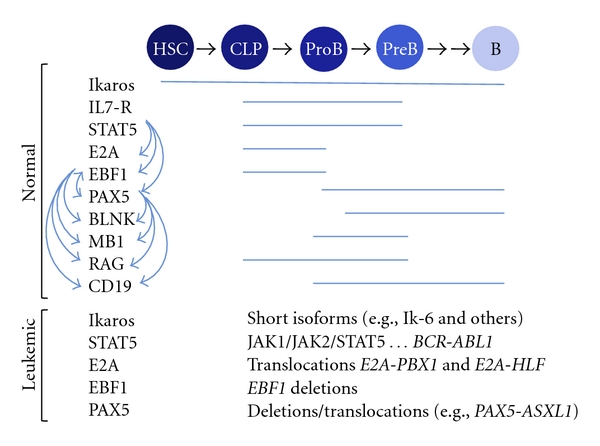
Homeostatic and leukemic expression of receptors, signaling proteins, and transcription factors along the B cell pathway. Developmental stages are indicated starting with the hematopoietic stem cell (HSC), the common lymphoid progenitor (CLP), and into the B cell pathway, stages proB, preB, and mature B cells. Normal gene expression along the developmental pathway is indicated with blue bars, and expression dependency between proteins is indicated with arrows. Most common modified forms of these receptors, signaling proteins, and transcription factors associated with acute lymphoblastic leukemia are also indicated.

High expression levels of the short Ikaros isoforms, particularly the dominant negative Ik-6, are also associated with high risk leukemia [100–105]. However, recent reports have showed that all Ikaros isoforms are expressed in patients with B ALL, although their level of expression differs [106]. Most of the BCR-ABL1^pos^ B ALL patients have deletions in *IKZF1* and increased levels of the short isoforms; however, Ik-6 has also been found to be elevated in BCR-ABL1^neg^ patients. In all these cases, deletion in the *IKZF1* gene is associated with a poor outcome; therefore, Ikaros isoform expression is an important prognostic factor in BCR-ABL1^pos^ and BCR-ABL1^neg^ B ALL [91, 107]. Elevated expression of Ik-6 is frequently correlated with elevated expression of Aiol-1 [105]. It has been proposed that the high level of Ikaros short isoform expression is due to genetic lesions. Supporting this idea, *IKZF1* somatic deletions have been found in up to 35% of recurrences [97] and in 23.9% of the patients with elevated levels of minimal residual disease [107]. Thereby, full-length Ikaros seems to be a leukemia suppressor that is inhibited by its dominant negative isoforms [101]. The currently available information on *IKZF3* expression in ALL is limited. However, Liippo et al. reported that the majority of the patients with ALL expressed several Aiolos isoforms [72]. A summary of homeostatic and leukemic expression of receptors, signaling proteins, and transcription factors along the B cell pathway is shown in Figure 3.

## 5. Conclusion

Genes critical for commitment to the B cell lineage and B cell maintenance are often mutated in B ALL. Genome-wide analysis methods that measure expression levels and assess the presence of genetic and epigenetic abnormalities in B cell ALL have recently provided new insights into the genetic lesions most frequently associated with this disease. These genetic signatures are paving the way for (1) a better understanding of the origin of B cell ALL, (2) a precise classification of the disease, and (3) accurate predictive profiles, which are especially necessary for those patients that lack a high-risk associated translocation but are prone to relapse. These data will also be useful for the generation of more effective drugs that act specifically to complement or counteract the genetic lesions associated with ALL. In the near future, such drugs will have a major impact on the overall cure rates [65, 66].
